# A Randomized Trial of At-Home COVID-19 Tests, Telemedicine, and Rapid Prescription Delivery for Immunocompromised Individuals

**DOI:** 10.1016/j.mayocpiqo.2025.100627

**Published:** 2025-05-22

**Authors:** Julia Moore Vogel, Ting-Yang Hung, Erin Coughlin, Felipe Delgado, Vik Kheterpal, Giorgio Quer, Eric Topol

**Affiliations:** a Scripps Research Translational Institute, Scripps Research, La Jolla, CA; b CareEvolution, Ann Arbor, MI

## Abstract

**Objective:**

To evaluate whether access to at-home COVID-19 tests, telemedicine, and same-day prescription delivery could reduce COVID cases, hospitalizations, and the cost of COVID care for the high-risk populations using a randomized controlled trial.

**Patients and Methods:**

Individuals participated remotely between December 1, 2022, and May 16, 2024, with half (n=346, 51.5%) receiving the option to access 10 at-home COVID-19 tests per month for themselves and others in their household as well as telemedicine and same-day Paxlovid delivery, and half (n=325, 48.4%) following their standard testing and treatment practices. Outcome data were collected from surveys, electronic health records and claims.

**Results:**

Intensive care unit admissions were significantly reduced for intervention participants vs control participants (0.3% vs 4.6%, n=1 vs 13; *P*<.001). Reported COVID case incidence did not differ significantly (19.0% vs 20.4%, n=59 vs 58; *P*=.69), nor did hospitalizations (5.2% vs 7.7%, n=15 vs 22; *P*=.14). The intervention was estimated to result in a reduction of $3650 in the cost of COVID care per person. The specific intervention used is no longer available in the market and alternatives should be considered. Evolution of SARS-CoV-2 could change the effect observed. Survey completion was higher in the intervention group.

**Conclusion:**

In immunocompromised individuals and those at least aged 65 years, access to at-home COVID tests, telemedicine, and rapid Paxlovid delivery reduced the severity of COVID-19 infections, as reflected by a reduced need for intensive care unit admissions; this has the potential to reduce the cost of COVID care.

**Trial Registration:**

clinicaltrials.gov Identifier: NCT05655546

Immunocompromised people and those who are aged at least 65 years have been at high-risk for adverse COVID-19 outcomes since the pandemic began.^1^, ^2^, ^3^ Immunocompromised individuals comprise 3% to 7% of the US population,^4^^,^^5^ and adults aged ≥65 years comprise 17%.^6^ COVID-19 hospitalization rates remain much higher for individuals aged ≥65 years than all other age groups.^7^ Vaccination against COVID-19 in immunocompromised individuals leads to less seroconversion than in immunocompetent populations, even after booster doses.^8^, ^9^, ^10^ The degree of seroconversion varies based on the nature of immunosuppression, which is correlated with adverse COVID-19 outcomes.^11^ Further, some immunocompromised individuals experience prolonged infection that can lead to the evolution of new variants that can resist existing therapies, become more transmissible, and/or become more pathogenic.^12^, ^13^, ^14^ Therefore, minimizing infection rate and duration in immunocompromised individuals is a public health priority. Earlier treatment of COVID-19 in high-risk individuals can improve outcomes.^15^

There are little data on the current level of risk of infection and adverse outcomes for these high-risk populations because most public health precautions have ceased, such as mandatory masking in health care settings and isolation requirements for individuals with active infections.^16^ We conducted a randomized controlled trial to determine whether supplying at-home COVID-19 tests, telemedicine, and rapid prescription delivery of Paxlovid, when indicated, could reduce the number, severity, and health care cost of COVID-19 infections in immunocompromised individuals and those who are aged ≥65 years.

## Patients and Methods

### Study Design

We conducted a randomized controlled trial to evaluate whether access to at-home COVID-19 tests, on-demand telemedicine, and same-day prescription delivery could reduce COVID cases and adverse outcomes in immunocompromised individuals. Half of the participants were randomized to be offered 10 Cue Health at-home COVID-19 nucleic acid amplification tests (NAATs), the molecular equivalent of a polymerase chain reaction (PCR) test, along with access to on-demand telemedicine and same-day prescription delivery, also provided by Cue Health (intervention arm). The other half of the patients were randomized to continue their normal COVID testing and treatment practices (control arm). Individuals in the intervention arm could use their study-provided COVID tests themselves, or give them to others in their household, to explore reducing within-household transmission. The study could not be blinded due to the nature of the intervention. All study activities were conducted remotely and in English, using CareEvolution’s MyDataHelps digital clinical trial platform and providing participant support by e-mail. Participants were recruited from across the United States through digital means, leveraging both paid and organic social media campaigns. Institutional review board approval was obtained from Scripps Health before the start of data collection, and the study was preregistered on clinicaltrials.gov (NCT05655546).^17^

### Participants

Inclusion criteria were as follows: (1) living in the United States (Supplemental Figure 1, available online at http://www.mcpiqojournal.org); (2) aged ≥18 years; (3) can read and understand English; (4) use of compatible broadband-connected device; (5) completion of at least the initial COVID-19 vaccination series; (6) willingness and ability to participate in study interventions including use of smartphone, camera and Bluetooth; upload verification of eligibility, if needed; complete surveys; use of Cue Health application; and use of MyDataHelps web or native application; and (7) belonging to 1 of these following 2 groups: (a) immunocompromised owing to disease or therapy, including symptomatic HIV, graft-vs-host disease, immunoglobulin deficiency/immunodeficiency, immunosuppressive therapy,^18^ leukemia, lymphoma (Hodgkin or non-Hodgkin), metastatic cancer, multiple myeloma, solid organ malignancy, hematopoietic stem cell transplant, and solid organ transplant, and/or (b) aged ≥65 years. There were no additional exclusion criteria. Eligibility criteria 1 through 6 were confirmed by eligibility survey responses; criterion 7 was validated through 1 of the following 3 ways: (1) using an automated analysis of claims or electronic health record (EHR) data shared by the participant via the MyDataHelps platform; (2) study staff review of information uploaded through the MyDataHelps portal; and (3) study staff review of information the participant shared by e-mail. All participants provided e-consent through the MyDataHelps portal, after being given the opportunity to contact study staff with any questions. All demographic data were self-reported.

### Randomization

Participants were randomly assigned to the control or intervention arm at the time they were confirmed to meet the eligibility criteria, using randomization with a 50% probability for each arm. Randomization was automated through the MyDataHelps platform and did not require study staff involvement. Study staff had access to the participant’s assigned arm only after randomization. Participants in the intervention arm were then invited to create a Cue Health account and place an order for a Cue Health reader and tests, at no cost to participants. Intervention participants who tested positive for COVID-19 were also offered access to on-demand telemedicine and rapid prescription delivery of Paxlovid, when indicated, from Cue Health. Participants were aware of their arm assignment. Other than intervention-specific tasks, participant experiences were identical between the 2 arms.

### Procedures

After becoming aware of the study, individuals were directed to the study landing page and then an eligibility survey. Eligible individuals were invited to create a MyDataHelps account (or log into an existing account) and begin the e-consent process. Following e-consent, participants were asked to complete a baseline survey (with demographic information; Supplemental Table 1, available online at http://www.mcpiqojournal.org), share health claims, and/or EHR data and confirm they met eligibility criteria based on age or immunocompromised status (via claims or EHR data or manual information sharing). Health claims sharing was required for individuals who were eligible for the study because of being ≥65 years of age because most individuals in the United States who are ≥65 years of age are able to share claims through the Center for Medicare and Medicaid Services portal; health claims sharing was not required for those who are otherwise immunocompromised.

Next, participants were randomized to control and intervention arms, with all participants being given the option to complete a survey about their COVID-19 experience in the past month, and intervention participants also being asked to create a Cue Health account, download the Cue Health application, and order a Cue Health reader and 10 tests, both manufactured in San Diego, CA. Use of the Cue Health tests and application also came with the option for no cost, on-demand telemedicine visits if one tested positive for COVID-19 and same-day prescription delivery (if ordered by 3 pm local time; otherwise, it would be delivered the following day), if indicated. Intervention participants were notified that they could seek care from other sources, based on their preference.

Going forward, participants had the option to complete a survey about their COVID-19 experiences any time and were prompted to do so once per month. On May 13, 2024, the Food and Drug Administration (FDA) released a communication that Cue Health tests should no longer be used owing to changes that Cue Health did not communicate to FDA.^19^ The study team shared that communication with participants and ended the study early. All participants were sent an end-of-study survey, asking about their COVID-19 experiences throughout the duration of their participation. Participant compensation was up to $115 in the form of Amazon gift cards: $20 for completing all baseline steps, $10 for each monthly survey completed, half of remaining monthly survey compensation for completing the end-of-study survey (eg, if the participant had not completed 3 monthly surveys, they would receive $15 for the end-of-study survey). When the study was concluding, we sought to increase the quantity of claims data collected and added a $15 incentive for claims sharing from those who had not previously shared claims data. All gift cards were distributed using the Amazon Application Programming Interface to deliver codes to participants within the MyDataHelps portal.

### Outcomes

The primary outcome measured was the number of hospitalizations (including admissions and intensive care unit [ICU] stays) and the estimated cost of care associated with COVID-19. We hypothesized that access to at-home tests, on-demand telemedicine, and rapid prescription delivery could lead to earlier detection and treatment of COVID-19 infection, which could improve outcomes. A secondary outcome was the number of reported COVID-19 cases. Both outcomes were measured using participant surveys, claims, and EHR data. Survey responses were used to count COVID medications, hospital admissions, and ICU stays (Supplemental Appendix, available online at http://www.mcpiqojournal.org, for full survey content and data). For claims and EHR data, COVID medications were defined as being prescribed: Paxlovid, molnupiravir, remdesivir, nirmatrelvir, and ritonavir. COVID hospital admissions were defined as claim type is “HOSP” or “Institutional” or location display contains “inpatient” with COVID diagnosis or COVID procedure or COVID medication in the same medical episode. COVID ICU was defined as ICU or critical care records with COVID diagnosis or COVID procedure or COVID medication in the same medical episode. Claims and EHR data were analyzed on August 1, 2024, about 3.5 months after enrollment closed on May 16, 2024. We analyzed survey data collected within an 8-month window from each participant’s study onboarding date or through August 1, 2024, whichever was shorter. Most participants were enrolled for over 8 months (n=533) (Supplemental Figure 2, available online at http://www.mcpiqojournal.org). We reported on the union of data, that is, if a hospitalization was reported in claims or survey data, it was included. We expected there could be missing data and that all 3 data sources are lower bounds of COVID-19 cases and hospitalizations.

### Cost of Care

To estimate the cost of COVID health care, we used data from FAIR Health,^20^ an independent nonprofit that collects data for and manages the nation’s largest database of privately billed health insurance claims, reporting the average cost of a COVID outpatient, COVID noncomplex inpatient, and COVID complex inpatient to approximate the cost of each COVID case with medication only, COVID hospitalization (non-ICU), and COVID ICU stay^20^ (Table 1). Each participant’s cost of care was one of these values, whichever was the costliest type of care they received. The estimated allowed amount was primarily reported because that was the amount paid by insurance carriers.

**Table 1:** Cost of COVID-19 Health Care, Based on Degree of Case Complexity

FAIR Health national average costs of COVID health care	Estimated allowed amount ($)	Charged amount ($)
COVID outpatient	1008	2557
COVID inpatient noncomplex	33,525	74,591
COVID inpatient complex	98,139	317,810

### Statistical Analyses

The study was designed to have a sample size of 10,000 participants with a minimum of 800 reported COVID-19 cases. We projected a 20% hospitalization rate among immunocompromised people who contracted COVID-19 and a 25% ICU rate among those who were hospitalized, based on COVID-19 hospitalization data reported through February 2022.^21^ The objective was to assess whether there was a difference in the probability of contracting COVID-19, hospitalization due to COVID-19, and ICU admission due to COVID-19, and estimated COVID health care cost, between individuals with standard of care (control) and with access to at-home COVID-19 tests, telemedicine, and rapid prescription Paxlovid delivery (intervention). We calculated the ratio of individuals who presented these outcomes in the 2 arms. Comparisons between the binary COVID-19 outcomes in the 2 groups were also tested for statistical significance (ie, *P*<.05) with a proportions χ^2^ test. The 2 groups were compared also in terms of average cost per participant, using a permutation test with 10,000 permutations. We did not use the standard *t* test because the data were not normally distributed. To check the balance between control and treatment cohorts, we used a 2-tailed χ^2^ test of independence. The statistical analysis was performed using publicly available statistical tools (python version 3.8.3, scipy version 1.10.1, and statsmodels version 0.14.1).

### Role of the Funding Sources

Cue Health provided funding for this work and collaborated with Scripps Research in study design and operationalization. Digital trial infrastructure was also supported by the National Center for Advancing Translational Sciences of the National Institutes of Health (NIH) under Award Number UM1TR004407. NIH’s only role was providing funding. The data analysis, interpretation, and manuscript preparation was conducted independently by Scripps Research and CareEvolution.

## Results

Recruitment began on December 1, 2022, and ended on May 16, 2024, resulting in 671 confirmed eligible and randomized participants across the United States (Figure, Supplemental Figure 1, available online at http://www.mcpiqojournal.org), statistically balanced between arms (Table 2). The study ended prematurely because changes in Cue Health’s protocols were not reported to the FDA, resulting in FDA advising that their COVID-19 tests not be used.^19^ Of the eligible participants, 304 were automatically verified as immunocompromised or at least 65 years old using claims or EHR data, and 367 were manually verified either by participants uploading or e-mailing documentation of eligibility for study coordinator review. Most diagnoses could be confirmed automatically for participants who shared EHR or claims data; however, manual verification by a study coordinator was often required for immunosuppressant medications, as well as for participants who did not have EHR or claims data available to share electronically. After being offered a $15 incentive to share claims data at the end of the study, only 1 additional participant shared claims data. This suggests that either most eligible individuals had already shared claims data or the incentive was insufficient to overcome any data sharing barriers. EHR data were collected following the Fast Healthcare Interoperability Resources standard from 129 end points. Of the 346 participants who completed the end-of-study survey, 25% (n=88) had not previously completed any monthly surveys; it is possible that the participants were motivated by the increased incentive or because it was their last chance to share information about their exposures to COVID-19 and any infections.

**Figure:**
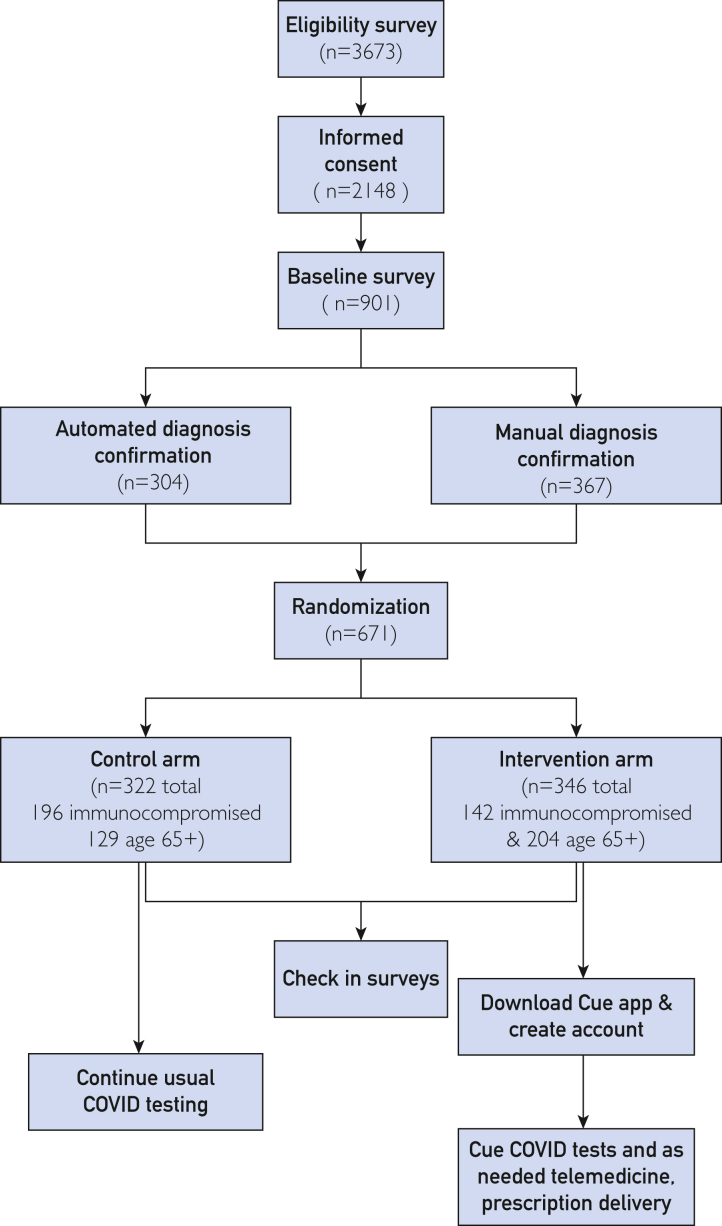
Participant enrollment and workflow. A summary of the steps between eligibility, randomization, and downstream activities and number of participants at each stage.

**Table 2:** Trial Profile

Category	Control	Treatment	All	*P*
No. of participants who completed the baseline survey	250 (46)	292 (54)	542	
Conditions				
High blood pressure	98 (39)	92 (32)	190 (35)	.061
Asthma	36 (14)	58 (20)	94 (17)	.090
Diabetes	30 (12)	33 (11)	63 (12)	.800
Chronic kidney disease	24 (10)	23 (8)	47 (9)	.477
Chronic obstructive pulmonary disease	8 (3)	10 (3)	18 (3)	.884
Heart failure	6 (2)	5 (2)	11 (2)	.571
Emphysema	5 (2)	5 (2)	10 (2)	.804
Chronic bronchitis	3 (1)	7 (2)	10 (2)	.302
Other	113 (45)	126 (43)	239 (44)	.632
No response	64 (26)	78 (27)	142 (26)	.769
Doses (No. of COVID-19 vaccines)				
1	1 (0)	0 (0)	1 (0)	
2	3 (1)	10 (3)	13 (2)	
3	16 (6)	29 (10)	45 (8)	
4	49 (20)	52 (18)	101 (19)	
5	88 (35)	100 (34)	188 (35)	
6	64 (26)	66 (23)	130 (24)	
7	23 (9)	25 (9)	48 (9)	
8	6 (2)	10 (3)	16 (3)	
Average doses taken	5.1	5.0	5.1	.290
Type of COVID-19 vaccine				
Full	632	761	1393	
Boost	652	705	1357	
Manufacturer of COVID-19 vaccine				
Moderna	616	749	1365	
Pfizer	630	686	1316	
Janssen	15	19	34	
Novavax	18	6	24	
Other	5	6	11	
Age bracket (y)				
18-25	3 (1)	5 (2)	8 (1)	
26-35	9 (4)	11 (4)	20 (4)	
36-45	21 (8)	26 (9)	47 (9)	
46-55	16 (6)	22 (8)	38 (7)	
56-65	23 (9)	24 (8)	47 (9)	
66-75	135 (54)	167 (57)	302 (56)	
76-85	39 (16)	35 (12)	74 (14)	
86+	4 (2)	2 (1)	6 (1)	
Average age	64.8	64.1	64.4	.524
Gender				
Female	150 (60)	198 (68)	348 (64)	.059
Male	94 (38)	90 (31)	184 (34)	.097
Nonbinary, trangender, or prefer not to answer	8 (3)	8 (3)	16 (3)	.752
Nonbinary	8 (3)	4 (1)	12 (2)	
Transgender	0 (0)	4 (1)	4 (1)	
Prefer not to answer	0 (0)	1 (0)	1 (0)	
Race				
White only	218 (87)	262 (90)	480 (89)	.357
Hispanic only	1 (0)	5 (2)	6 (1)	.146
Asian only	8 (3)	8 (3)	16 (3)	.752
Black/AA only	7 (3)	3 (1)	10 (2)	.126
AI/AN, ME, NH, and other races	5 (2)	1 (0)	6 (1)	.066
AI/AN only	0 (0)	0 (0)	0 (0)	
ME only	1 (0)	0 (0)	1 (0)	
NH only	0 (0)	0 (0)	0 (0)	
Other only	4 (2)	1 (0)	5 (2)	
Multiple races	8 (3)	12 (4)	20 (4)	.576
Prefer not to answer	3 (1)	1 (0)	4 (1)	.2445
None of these fully describe me	0 (0)	0 (0)	0 (0)	NA

Values are n (%) or n unless specified. Participant characteristics from the baseline survey: of the 671 participants (control=325, treatment=346), 542 participants completed the baseline survey. A participant can have more than 1 response to some questions. When χ^2^ test was used to examine whether the control and treatment distributions were independent, *P* values were all >.05, indicating that the cohorts were adequately balanced.

AA, African American; AI/AN, American Indian/Alaskan Native; ME, Middle Eastern; NH, Native Hawiian.

Although we aimed to enroll participants who were representative of the US population, limited outreach funding resulted in lower engagement of Black, Indigenous, and People of Color (often abbreviated BIPOC) participants (Table 2). Otherwise participant demographic characteristics reflected the population of interest. All attributes examined were statistically determined to be balanced between control and intervention populations (ie, *P*>.05), including race/ethnicity, gender, age, COVID vaccination status and type, health conditions, and insurance type. The study team was not made aware of any adverse events. The average duration of study participation was 367 days, about 12 months (Supplemental Figure 2).

Outcome data were drawn from 4 sources: monthly surveys, an end-of-study survey (covering the entire time a participant was enrolled), claims data, and EHR data (Supplemental Tables 2-6, available online at http://www.mcpiqojournal.org). Monthly and end-of-survey data responses were completed more frequently in the intervention arm (55% of respondents as opposed to 45% from the control arm). We hypothesized this was due to the presence of study-provided tests reminding participants of their participation in the study more often. It is possible this led to an overestimation of control arm COVID cases and cost of care in the survey data. We report results as a percentage of responses. The frequencies of claims and EHR data connections were consistent between arms (81.5% and 80.6% in control and intervention arms, respectively). As a result of Cue Health’s (the funding company) bankruptcy, the data for proportion of participants in the intervention group who had a telemedicine consult or home delivery of Paxlovid were unavailable, as was the number of test orders placed by each intervention participant. We display subgroup data for immunocompromised individuals and those who were 65 years or older and not otherwise immunocompromised because their underlying risk levels may differ.

The need for admission to the ICU reduced significantly in the intervention arm, relative to the control arm (0.3% and 4.6%; difference, 4.3 percentage points [pp]; 95% CI, 1.7-6.7 pp; *P*<.001) (Table 3). The odds ratio of an ICU admission was 14.8 in the control group relative to the intervention group (95% CI, 1.92-113.6). The highest ICU admission rate was among immunocompromised participants, at 5.6% of control participants, compared with 3.2% of control participants aged 65 years and older. The differences in the rates of being prescribed COVID medications (19.0% and 20.4%; *P*=.69; 95% CI, −6.74 to 5.08 pp difference between intervention and control arms) and COVID hospital admissions (5.2% and 7.7%; *P*=.14; 95% CI, −1.03 to 6.79 pp difference between intervention and control arms), were not statistically significant (Table 3). COVID cases were not consistently available in claims or EHR data; therefore, we used COVID prescriptions as the closest approximation, which were consistently available because individuals who met the study’s eligibility criteria were eligible for antivirals to treat COVID infections. The differences in types of medications prescribed were also not statistically significant between control and intervention arms (*P*=.13) (Supplemental Table 7, available online at http://www.mcpiqojournal.org). The estimated cost of COVID care was significantly higher in the control group relative to that of the intervention group ($5740 and $2022, respectively; average difference, $3718; 95% CI, $1085-$6351; *P*=.005) (Table 4). The average estimated cost of COVID care was 2.7 times higher for control vs intervention participants (Supplemental Table 8, available online at http://www.mcpiqojournal.org). Data from individual sources showed similar trends (Supplemental Tables 2-6). Although we did not reach our desired sample size, owing to the intervention becoming unavailable, the intervention’s effect size was larger than expected, and the smaller sample size was sufficient.

**Table 3:** COVID Medication, Hospital Visits, and ICU Stays Throughout the Study

Participant category	No. of patients with claims and/or survey data	Prescribed COVID medication, n (%)	COVID hospital admission, n (%)	COVID ICU stay, n (%)
Control	285	58 (20.4)	22 (7.7)	13 (4.6)
65+ y	125	23 (18.4)	7 (5.6)	4 (3.2)
Immunocompromised	160	35 (21.9)	15 (9.4)	9 (5.6)
Intervention	310	59 (19.0)	15 (4.8)	1 (0.3)
65+ y	134	22 (16.4)	4 (3.0)	0 (0.0)
Immunocompromised	176	37 (21.0)	11 (6.3)	1 (0.6)
Total	595	117 (19.7)	37 (6.2)	14 (2.4)

ICU, intensive care unit.

**Table 4:** Estimated Average Cost of COVID Care Based on Outcomes Reported in Table 3 and FAIR Health Costs of COVID Care (Table 1 and Supplemental Table 7)^20^

Average estimated cost of care	Charged amount ($)	Allowed amount ($)
Control	17,372	5740
65+ y	12,431	4131
Immunocompromised	21,233	6998
Intervention	4880	2022
65+ y	2646	1166
Immunocompromised	6581	2674
Entire cohort	10,864	3803
Difference—65+ y	9784	2964
Difference—immunocompromised	14,652	4324
Difference—average	12,492	3718

## Discussion

In this trial, providing individuals at high-risk of adverse COVID-19 outcomes with at-home COVID tests, on-demand telemedicine, and rapid prescription delivery reduced the number of ICU stays, which reduced the estimated cost for COVID-19 health care per person. This trial also characterized the ongoing effect of the COVID-19 pandemic on high-risk populations: on average, in the group experiencing standard of care during a 1-year study, 5.6% of immunocompromised participants experienced a COVID ICU stay and 3.2% of individuals aged 65 years or older did. As a result, the cost of COVID-19 health care during the study was estimated to be an average of $3718 higher per control participant, compared with that of intervention participants. At an estimated average of $286 per month ($333 for immunocompromised participants), this was more than the cost of 5 Cue Health tests per month (which were about $50 each) and much higher than the cost of supplying high-risk individuals with daily antigen COVID-19 tests (which cost about $6 each, or $180 for 30 tests, at the time of writing the manuscript). The expected increased cost of implementing on-demand telemedicine and rapid prescription delivery compared with that of current practices might vary per health system, but Cue charged $99 for this service. Further, alongside daily antigen testing, we estimated that $1272 per individual per year would remain for these costs before implementing the intervention became more financially costly than not implementing it. In addition to cost savings at the individual level, there is a potential for the intervention to help reduce the risk of prolonged infections, thereby reducing the emergence of new variants.^12^, ^13^, ^14^ This study adds to the evidence that public health and individual precautions are still warranted and cost-effective in 2024, when both COVID-19 data collection and public health measures have been drastically reduced compared with those earlier in the pandemic.

We hypothesized that the reduction in ICU stays is due to intervention individuals accessing a positive test result and treatment earlier in the course of their infection. The hospitalization rate we observed was consistent with previous findings.^2^^,^^21^ The intervention did not affect the rate of participants contracting COVID-19, despite that tests could be shared with others in the participant’s household, or which drug was prescribed to treat COVID infections. This could be due to infections coming from outside the household, a lack of willingness for others in the household to test, or the study participant using all tests themselves. The intervention did not statistically significantly affect hospitalization rates, although there were directionally fewer hospitalizations within the intervention group. One possible explanation is that rapid detection and treatment of an infection is insufficient to prevent hospitalization in this population, indicating that infection prevention efforts should be prioritized.

The test used was an at-home NAAT, a molecular equivalent to PCR. Although, in general, NAATs are more sensitive than antigen tests, it is possible that antigen tests could be used when individuals have a fever with minimal loss of sensitivity.^22^ When an individual is aware they have been exposed to COVID-19 and they are not febrile, a NAAT or PCR test would improve sensitivity.^22^, ^23^, ^24^ This is a reason it is crucial to maintain community access to NAAT and/or PCR tests. However, accessing NAAT and PCR tests usually require leaving one’s home, and it is unknown how that requirement would affect testing uptake. It is also unknown what level and type of access to testing the control group had; about a quarter of the way through the study, the public health emergency portion of the pandemic ended,^25^ insurance carriers were no longer required to reimburse for at-home antigen tests, and the number of locations providing PCR testing declined.

It is not possible to separate the 3 components of the intervention arm—at-home tests, telemedicine, and rapid Paxlovid delivery—to determine a driving factor, and a lack of availability of data for the latter 2 components represents a limitation. Although the study was closed early owing to causes outside of the control of the authors, the data collected before closure were sufficient to drive the conclusions presented in this article. The FDA’s removal of device approval was primarily due to concern about device stability; although concern was expressed about the potential for this to affect the accuracy of results, there was no evidence presented that accuracy was affected. Our study was also limited by a lower-than-intended sample size and underrepresentation of Black, Indigenous, and people of color participants. Further, we saw higher study engagement in the intervention arm. This only affected survey completion data; the claims and EHR data were without bias between arms. The implication of this biased participation on survey data is not immediately clear; it could be that control data were underreported in the surveys (in which case, our results likely underestimated the intervention’s effect) or that control participants were more likely to complete a survey only when they had a COVID-19 infection. It is also possible that provider prescription practices varied, and we were unable to detect this. Further, the test, telemedicine, and prescription delivery offering that was used for this study is no longer available, and there is not a clear replacement at-home test with the same level of specificity. It is possible that individuals in the intervention arm changed their behavior as a result of having tests available, for example, by doing activities with a higher risk of contracting COVID-19. The cost of the molecular tests used in this trial is high, and there is potential for marked reduction of rapid tests in the future, making the intervention even more alluring from a benefit-to-cost ratio standpoint. Because this intervention did not reduce infection rates, a complementary option is providing high quality masks, for example, N95 masks, in particular to those at high-risk and, potentially more broadly, to proactively reduce infection rates.

## Conclusion

Future research should include a similar study evaluating each component of the intervention (ie, at-home tests, telemedicine, and rapid Paxlovid delivery), along with a comparison of PCR and antigen tests. In particular, it would be helpful to analyze when someone was exposed to COVID-19 (if known), when symptoms began, when they tested positive, and when they began antivirals; this can improve understanding of the role of rapid detection and intervention in improving outcomes. We recommend efforts to increase adoption of testing, enabling earlier detection. For example, tests could be sent to participants on a regular basis by default, to ensure they are readily available when needed. Educating participants that early testing and treatment can reduce their risk of adverse outcomes may improve testing uptake and could improve testing uptake among their close contacts. Future studies may include wearable devices, which can detect changes from one’s personalized baseline that suggest a viral infection and suggest that participants test.^26^^,^^27^ The optimal number of tests to be provided within a given timeframe is another variable that should be explored. For example, participants may benefit from having a certain size stockpile to feel comfortable sharing tests with others to reduce the COVID-19 case rate. Making Paxlovid and other COVID-19 treatments easier to obtain, for example, without requiring insurance preauthorization, may also improve outcomes. On the basis of these results and other data demonstrating the ongoing risk of COVID-19 to high-risk individuals,^2^^,^^21^ we recommend that payers and public health organizations provide COVID tests and rapid treatment to high-risk individuals, at no cost, for as long as the virus continues to circulate.

## Potential Competing Interests

Dr Kheterpal is an employee and shareholder of CareEvolution. All other authors report no competing interests.

## Ethics Statement

This work was reviewed and approved by the Scripps institutional review board. Signed informed consent was obtained from all participants.
