# Supplementary material for: A Randomized Trial of At-Home COVID-19 Tests, Telemedicine, and Rapid Prescription Delivery for Immunocompromised Individuals
Source: Mayo Clin Proc Innov Qual Outcomes. 2025 May 22;9(3):100627. doi: 10.1016/j.mayocpiqo.2025.100627 (PMC12152619; doi:10.1016/j.mayocpiqo.2025.100627)
Supplement: Supplemental Materials [file mmc1.docx]

# Supplemental Materials

A randomized trial of at-home COVID-19 tests, telemedicine, and rapid prescription delivery for immunocompromised individuals

## Authors

Julia Moore Vogel^1^, Ting-Yang Hung^1^, Erin Coughlin^1^, Felipe Delgado^1^, Vik Kheterpal^2^, Giorgio Quer^1^, Eric Topol^1^

^1^ Scripps Research Translational Institute

^2^ CareEvolution

[**Supplemental Materials 1**](#_lejp5oj5auwc)

[Authors 1](#_4wwofvw9en58)

[Supplemental Tables 2](#_aagvgs80wzpk)

[Participant Characteristics 2](#_8no391g1lkcw)

[Supplemental Table 1 4](#_wa9wv7r2239o)

[Outcome data from each source 5](#_hw3nvvi004kl)

[Supplemental Table 2 5](#_uh6qbynax8mj)

[Supplemental Table 3 5](#_1te6kaeqgpbv)

[Supplemental Table 4 6](#_q7ckb1251t9v)

[Supplemental Table 5 6](#_shl9q8co70ga)

[Supplemental Table 6 7](#_gx74qewqroic)

[Supplemental Table 7 8](#_a1mze0lnoupj)

[Supplemental Table 8 9](#_vw9c0jsbwvgq)

[Supplemental Figures 10](#_q05i6h1zqadl)

[Supplemental Figure 1 10](#_3u60fxvt9azi)

[Supplemental Figure 2 11](#_7evqs2elw9pp)

[Surveys 11](#_vdw1zypev396)

[Baseline Survey 11](#_1atssf8i9vir)

[Check-in Survey 12](#_hfjgq33u05ul)

[Data Dictionary 13](#_epmd7jqno7fx)

##

## Supplemental Tables

### Outcome data from each source

Outcome data are obtained from the union of claims, EHR, and survey data, i.e. if one data source shows a hospitalization, we include that hospitalization in the count and if multiple sources for one participant show a hospitalization, we include that as a count of one hospitalization. Below we show individual data sources and combined data so that a reader can see the general trends of more ICU stays in the control group and similar numbers of cases and COVID hospitalizations are present in each individual source as well as the aggregate data.

|  | Control | Treatment |
| --- | --- | --- |
| Total participants | 325 | 346 |
| Survey data only | 20 | 31 |
| Claims data only | 64 | 48 |
| Have both survey and claim data | 201 | 231 |
| Have neither survey nor claim data | 40 | 36 |

|  | Control | Treatment | Total |
| --- | --- | --- | --- |
| Medicaid or Medicare | 179 | 185 | 364 |
| Private insurance | 77 | 89 | 166 |
| Both | 9 | 5 | 14 |

#### Supplemental Table 1

Sources of outcomes data, insurance type, and number of participants with data from each source within each arm.

|  | # with claims or EHR | COVID med | | Any hospital admission | | COVID and non-ICU admission | | Any ICU | | COVID ICU | |
| --- | --- | --- | --- | --- | --- | --- | --- | --- | --- | --- | --- |
| **Control** | 265 | 30 | 11.3% | 64 | 24.2% | 9 | 3.4% | 13 | 4.9% | 6 | 2.3% |
| 65+ | 123 | 12 | 9.8% | 23 | 18.7% | 3 | 2.4% | 4 | 3.3% | 2 | 1.6% |
| IC | 142 | 18 | 12.7% | 41 | 28.9% | 6 | 4.2% | 9 | 6.3% | 4 | 2.8% |
| **Treatment** | 279 | 29 | 10.4% | 65 | 23.3% | 13 | 4.7% | 10 | 3.6% | 1 | 0.4% |
| 65+ | 131 | 13 | 9.9% | 17 | 13.0% | 4 | 3.1% | 3 | 2.3% | 0 | 0.0% |
| IC | 148 | 16 | 10.8% | 48 | 31.1% | 9 | 6.1% | 7 | 4.7% | 1 | 0.1% |
| **Total** | 544 | 59 | 10.8% | 129 | 23.7% | 22 | 4.0% | 23 | 4.2% | 7 | 1.3% |

#### Supplemental Table 2

Outcomes from claims and EHR data; note immunocompromised is abbreviated IC.

|  | # with at least one monthly survey completed | COVID med | | COVID hospital visit (ER, and all admissions) | | COVID ICU | |
| --- | --- | --- | --- | --- | --- | --- | --- |
| **Control** | 197 | 32 | 16.2% | 10 | 5.1% | 7 | 3.6% |
| 65+ | 88 | 13 | 14.8% | 4 | 4.5% | 2 | 2.3% |
| IC | 109 | 19 | 17.4% | 6 | 5.5% | 5 | 4.6% |
| **Treatment** | 237 | 31 | 13.1% | 11 | 4.6% | 0 | 0.0% |
| 65+ | 99 | 10 | 10.1% | 4 | 4.0% | 0 | 0.0% |
| IC | 138 | 21 | 15.2% | 7 | 5.1% | 0 | 0.0% |
| **Total** | 434 | 63 | 14.5% | 21 | 4.8% | 7 | 1.6% |

#### Supplemental Table 3

Outcomes from monthly surveys

|  | # completed end of study surveys | COVID med | | COVID hospital visit (ER, and all admissions) | | COVID ICU | |
| --- | --- | --- | --- | --- | --- | --- | --- |
| **Control** | 146 | 24 | 16.4% | 3 | 2.1% | 1 | 0.7% |
| 65+ | 66 | 9 | 13.6% | 1 | 1.5% | 0 | 0.0% |
| IC | 80 | 15 | 18.8% | 2 | 2.5% | 1 | 1.3% |
| **Treatment** | 203 | 29 | 14.3% | 4 | 2.0% | 0 | 0.0% |
| 65+ | 89 | 10 | 11.2% | 0 | 0.0% | 0 | 0.0% |
| IC | 114 | 19 | 16.7% | 4 | 3.5% | 0 | 0.0% |
| **Total** | 347 | 53 | 15.3% | 7 | 2.0% | 1 | 0.3% |

#### Supplemental Table 4

Outcomes from end of study surveys

|  | # completed end of study surveys | COVID med | | COVID hospital visit (ER, and all admissions) | | COVID ICU | |
| --- | --- | --- | --- | --- | --- | --- | --- |
| **Control** | 221 | 40 | 18,1% | 12 | 5.4% | 8 | 3.6% |
| 65+ | 101 | 15 | 14.9% | 4 | 4% | 2 | 2.0% |
| IC | 120 | 25 | 20.8% | 8 | 6.7% | 6 | 5.0% |
| **Treatment** | 262 | 42 | 16.0% | 14 | 5.3% | 0 | 0.0% |
| 65+ | 108 | 15 | 13.9% | 4 | 3.7% | 0 | 0.0% |
| IC | 154 | 27 | 17.5% | 10 | 6.5% | 0 | 0.0% |
| **Total** | 483 | 82 | 17.0% | 26 | 5.4% | 8 | 1.7% |

#### Supplemental Table 5

Combined outcomes from monthly and end of study surveys

####

|  | Total | Exposed | COVID case | COVID med | Urgent care visit | ER visit | Hospital admission | ICU stay | Additional care |
| --- | --- | --- | --- | --- | --- | --- | --- | --- | --- |
| **Control** | 221 | 84 | 52 | 40 | 16 | 6 | 5 | 8 | 15 |
| 65+ | 101 | 39 | 23 | 15 | 5 | 4 | 2 | 2 | 9 |
| IC | 120 | 45 | 29 | 25 | 11 | 2 | 3 | 6 | 6 |
| **Treatment** | 262 | 114 | 58 | 42 | 14 | 10 | 4 | 0 | 14 |
| 65+ | 108 | 42 | 24 | 15 | 4 | 3 | 1 | 0 | 6 |
| IC | 154 | 72 | 34 | 27 | 10 | 7 | 3 | 0 | 8 |

#### Supplemental Table 6

Data from all participant surveys, including both monthly and end of study surveys. Of the 671 participants (control=325, treatment=346), 483 participants (control = 221, treatment = 262) completed at least one survey (monthly and end of study surveys). The descriptives below are the breakdown of participants who had ever indicated to (responded “Yes” to the question) experience the following conditions (see Check in survey below for questions). A participant could complete multiple surveys at different points in time. The descriptive only considers whether a participant had ever experienced the conditions regardless of the number of occurrences. Note that the COVID cases, medications, urgent care visits, hospitalization admissions, and needs for additional care are similar amongst control and treatment groups, but there are more ER visits amongst the treatment group and ICU stays amongst the control group.

|  | Any COVID med | Molnupiravir Only | Nirmaterlvir and Paxlovid | Paxlovid Only | Remdesivir Only | COVID ICU |
| --- | --- | --- | --- | --- | --- | --- |
| **Control** | 30 | 5 (16.7%) | 13 (43.3%) | 11 (36.7%) | 1 (3.3%) | 6 (2.3%) |
| 65+ | 12 | 1 (8.3%) | 7 (58.3%) | 3 (25.0%) | 1 (8.3%) | 2 (1.6%) |
| IC | 18 | 4 (22.2%) | 6 (33.3%) | 8 (44.4%) | 0 (0.0%) | 4 (2.8%) |
| **Treatment** | 29 | 2 (6.9%) | 21 (72.4%) | 6 (20.7%) | 1 (3.4%) | 1 (0.4%) |
| 65+ | 13 | 0 (0.0%) | 11 (84.6%) | 2 (15.4%) | 0 (0.0%) | 0 (0.0%) |
| IC | 16 | 2 (12.5%) | 10 (62.5%) | 4 (25.0%) | 0 (0.0%) | 1 (0.1%) |
| Total | 59 | 7 (11.9%) | 34 (57.6%) | 17 (28.8%) | 1 (1.7%) | 7 (1.3%) |

#### Supplemental Table 7

COVID medications and ICU stays based on claims and EHR data. Note: Paxlovid is a combination of Nirmaterlvir and Retonivir and there were no instances of Retonivir alone.

| **Cost of COVID care per individual** | | | No COVID case | Prescribed COVID medication | Hospitalization (non-ICU) | ICU stay |  |
| --- | --- | --- | --- | --- | --- | --- | --- |
| Estimated allowed costs | |  | 0 | 1,008 | 33,525 | 98,139 |  |
| Charged costs | |  | 0 | 2,557 | 74,591 | 317,810 |  |
|  | | | | | | | |
|  | | Number of ppts with data | No COVID case | Prescribed COVID medication | Hospitalization (non-ICU) | ICU stay |  |
| **Control** | | 285 | 205 | 58 | 9 | 13 |  |
| 65+ | | 125 | 95 | 23 | 3 | 4 |  |
| IC | | 160 | 110 | 35 | 6 | 9 |  |
| **Treatment** | | 310 | 236 | 59 | 14 | 1 |  |
| 65+ | | 134 | 108 | 22 | 4 | 0 |  |
| IC | | 176 | 128 | 37 | 10 | 1 |  |
| **Total** | | 595 | 441 | 117 | 23 | 14 |  |
|  | | | | | | | |
| ***Estimated allowed costs*** | |  |  |  |  |  | Total across cohort |
| **Control** | | 285 | 0 | 58,464 | 301,725 | 1,275,807 | 1,635,996 |
| 65+ | | 125 | 0 | 23,184 | 100,575 | 392,556 | 516,315 |
| IC | | 160 | 0 | 35,280 | 201,150 | 883,251 | 1,119,681 |
| **Treatment** | | 310 | 0 | 59,472 | 469,350 | 98,139 | 626,961 |
| 65+ | | 134 | 0 | 22,176 | 134,100 | 0 | 156,276 |
| IC | | 176 | 0 | 37,296 | 335,250 | 98,139 | 470,685 |
| **Total** | | 595 | 0 | 117,936 | 771,075 | 1,373,946 | 2,262,957 |
|  | | | | | | | |
| ***Charged costs*** | |  |  |  |  |  | Total across cohort |
| **Control** | | 285 | 0 | 148,306 | 671,319 | 4,131,530 | 4,951,155 |
| 65+ | | 125 | 0 | 58,811 | 223,773 | 1,271,240 | 1,553,824 |
| IC | | 160 | 0 | 89,495 | 447,546 | 2,860,290 | 3,397,331 |
| **Treatment** | | 310 | 0 | 150,863 | 1,044,274 | 317,810 | 1,512,947 |
| 65+ | | 134 | 0 | 56,254 | 298,364 | 0 | 354,618 |
| IC | | 176 | 0 | 94,609 | 745,910 | 317,810 | 1,158,329 |
| **Total** | | 595 | 0 | 299,169 | 1,715,593 | 4,449,340 | 6,464,102 |
|  | | | | | | | |
|  | | Entire study (12 months) | | Per month | |  | |
| Average estimated cost of care | | Charged amount | Allowed amount | Charged amount | Allowed amount |  |  |
| **Control** | | 17,372 | 5,740 | 1,336 | 442 |  |  |
| 65+ | | 12,431 | 4,131 | 956 | 318 |  |  |
| IC | | 21,233 | 6,998 | 1,633 | 538 |  |  |
| **Treatment** | | 4,880 | 2,022 | 375 | 156 |  |  |
| 65+ | | 2,646 | 1,166 | 204 | 90 |  |  |
| IC | | 6,581 | 2,674 | 506 | 206 |  |  |
| **Entire cohort** | | 10,864 | 3,803 | 836 | 293 |  |  |
| Difference between control and treatment: 65+ | | 9,784 | 2,964 | 753 | 228 |  |  |
| Difference between control and treatment: IC | | 14,652 | 4,324 | 1,127 | 333 |  |  |
| Difference between control and treatment: all | | 12,492 | 3,718 | 961 | 286 |  |  |

#### Supplemental Table 8

Cost of COVID health care calculations.

## Supplemental Figures


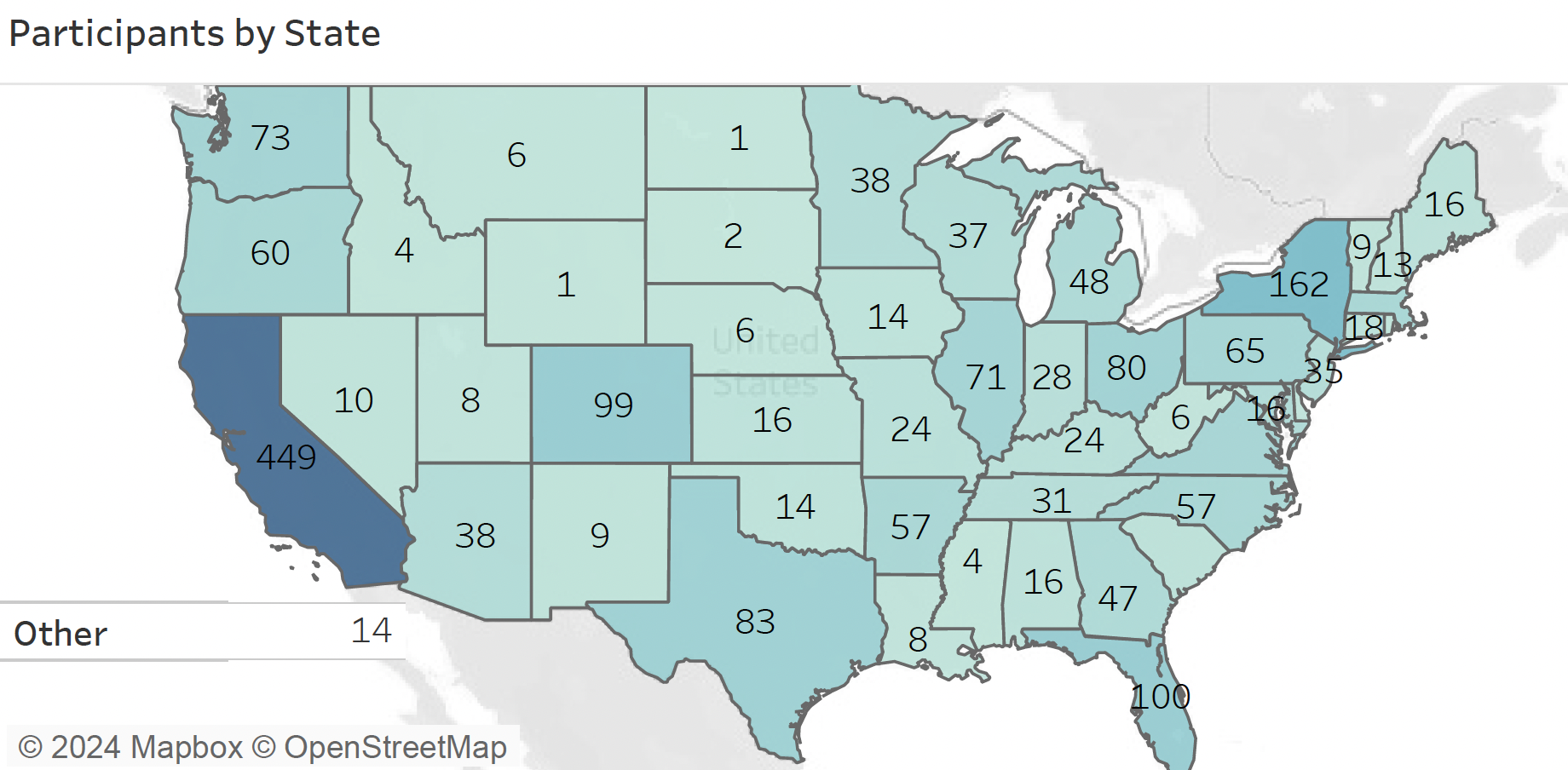


#### Supplemental Figure 1

Numbers of participants who completed consent per state.


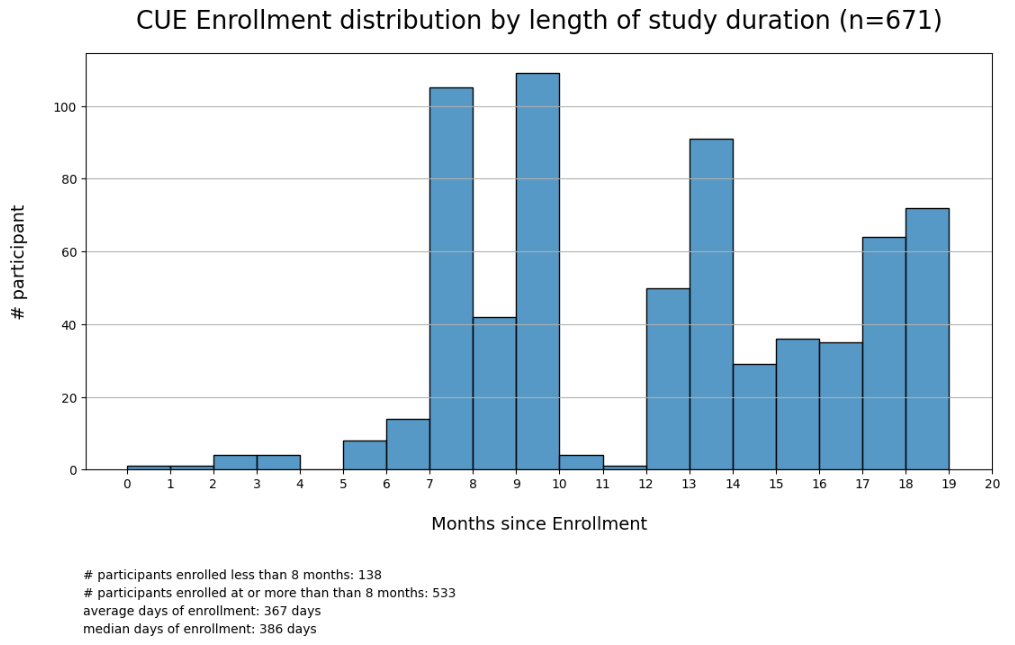


#### Supplemental Figure 2

Enrollment by study duration (months)

## Surveys

### Baseline Survey

1. What is your date of birth?
2. What is your phone number?
3. In case we cannot contact you, please provide the telephone number of a relative or friend who would know where you can be reached. This is optional.
4. What terms best express how you describe your gender identity? (Check all that apply)
   - Man
   - Woman
   - Transgender
   - Non Binary
   - Other (please specify)
   - Prefer not to answer
5. Which categories describe you? Select all that apply. Note, you may select more than one group
   - American Indian or Alaskan Native
   - Asian
   - Black, African American, African
   - Hispanic, Latino, or Spanish
   - Middle Eastern or Northern African
   - Native Hawaiian or other Pacific Islander
   - White
   - None of these fully describe me (please specify)
   - Prefer not to answer
6. What is your annual household income from all sources?
   - $25,000 or less
   - More than $25,000
7. What is your zip code?
8. Please indicate if you have any of the following chronic conditions
   - Diabetes
   - High blood pressure
   - Asthma
   - COPD
   - Emphysema
   - Chronic Bronchitis
   - Heart Failure
   - Chronic Kidney Disease
   - Other (please specify)
9. How many doses of the COVID-19 vaccine have you received?

If none: please confirm your medical exemption from COVID-19 vaccination was reviewed by the study team.

1. For your *[nth]* dose, please indicate:
   - Date
   - Type: [ ] Full dose [ ] Booster
   - Manufacturer:

[ ] Pfizer

[ ] Moderna

[ ] Janssen (Johnson & Johnson)

[ ] Novavax

[ ] Other (if you received it abroad)

[Repeats for the number of doses reported in 7.]

### Check-in Survey

Preamble varies based on when the survey is taken:

- At baseline: Over the last month have you:
- Monthly: Since we last checked in, have you:
- At end of study: Since you enrolled in ImmunoCARE, have you:
  - Been exposed to a close contact with COVID-19? [ ] Yes [ ] No
  - Contracted COVID-19? [ ] Yes [ ] No
  - Been prescribed medication for COVID-19? [ ] Yes [ ] No
  - Been to Urgent Care due to COVID-19? [ ] Yes [ ] No
  - Been to the Emergency Room due to COVID-19? [ ] Yes [ ] No
  - Been admitted to the hospital for COVID-19? [ ] Yes [ ] No
  - Been in the Intensive Care Unit due to COVID-19? [ ] Yes [ ] No
  - Needed additional health care (e.g. dialysis, CT scan, MRI) as a result of COVID-19? [ ] Yes [ ] No

Is there any context you would like to share about your previous responses? (open text box)

## Data Dictionary

The table below outlines the fields that are available upon request for the 671 participants enrolled in the study. Each row in the dataset corresponds to a participant.

| **Field** | **Type** | **Description** |
| --- | --- | --- |
|  |  |  |
| **Important Statuses General Info** |  |  |
| PID | Number | The de-identified participant identifier. |
| Race | String | The race of which Participants report in the baseline survey |
| Income level | String | The income level of which Participants report in the baseline survey |
| Gender | String | The gender of which Participants report in the baseline survey |
| Study arm | String | The group (control or intervention) of which participants are assigned to the study |
| Immunocompromised status | Binary | Whether participants are Immunocompromised due to disease or therapy.  Confirmation of immunocompromised status will be performed in one of three ways. Participants will be able to:   1. Upload a document directly into the MyDataHelps app 2. email it to a study coordinator OR 3. have their eligibility automatically verified via claims data.   Methods 1 and 2 will require the study team to review. |
| 65+ status | Binary | Whether participants are 65+ y/o |
|  |  |  |
| **Monthly survey and EoS** |  |  |
| Completed at least one survey | Binary | Whether participants completed at least one monthly survey or EoS |
| Exposed in survey | Binary | Whether participants report to have been exposed to COVID-19 at least once in the monthly survey or the EoS. |
| Contracted in survey | Binary | Whether participants report to have been contracted to COVID-19 at least once in the monthly survey or the EoS. |
| COVID medication in survey | Binary | Whether participants report to have been prescribed medication for COVID-19 at least once in the monthly survey or the EoS. |
| Urgent care in survey | Binary | Whether participants report to have been to Urgent Care due to COVID-19 at least once in the monthly survey or the EoS. |
| Emergency room in survey | Binary | Whether participants report to have been to the Emergency Room due to COVID-19 at least once in the monthly survey or the EoS. |
| Admitted to the hospital in survey | Binary | Whether participants report to have been admitted to the hospital for COVID-19 at least once in the monthly survey or the EoS. |
| ICU in survey | Binary | Whether participants report to have been to the Intensive Care Unit due to COVID-19 at least once in the monthly survey or the EoS. |
| Additional care in survey | Binary | Whether participants report to have needed additional health care (e.g. dialysis, CT scan, MRI) as a result of COVID-19 at least once in the monthly survey or the EoS. |
|  |  |  |
| **Claim** |  |  |
| Claim available | Binary | Whether participants agree to share the Claim record for the study participant and have at least one record in Claim |
| Covid medication in Claim | Binary | Whether participants have a record of COVID medication record in Claim during the study period |
| COVID diagnosis in Claim | Binary | Whether participants have a record of COVID-related diagnosis in Claim during the study period |
| COVID procedure in Claim | Binary | Whether participants have a record of COVID-related procedure in Claim during the study period |
| Hospital admission in Claim | Binary | Whether participants have a record of hospitalization in Claim during the study period |
| ICU in Claim | Binary | Whether participants have an ICU record in Claim during the study period |
